# The association of appendicular skeletal muscle mass with anthropometric, body composition, nutritional, inflammatory, and metabolic variables in non-dialysis-dependent chronic kidney disease men

**DOI:** 10.3389/fmed.2024.1380026

**Published:** 2024-07-25

**Authors:** Katarzyna Romejko, Katarzyna Szamotulska, Aleksandra Rymarz, Rozmyslowicz Tomasz, Stanisław Niemczyk

**Affiliations:** ^1^Department of Internal Diseases, Nephrology and Dialysis, Military Institute of Medicine—National Research Institute, Warsaw, Poland; ^2^Department of Epidemiology and Biostatistics, Institute of Mother and Child, Warsaw, Poland; ^3^Department of Pathology and Laboratory Medicine, Perelman School of Medicine, University of Pennsylvania, Philadelphia, PA, United States

**Keywords:** skeletal muscle mass, muscle atrophy, anthropometric measurements, body composition, chronic kidney disease

## Abstract

**Background:**

Muscle atrophy affects more than 50% of patients with chronic kidney disease (CKD) and is associated with increased morbidity and mortality. It is crucial to understand the mechanisms involved in the muscle atrophy in CKD and search for specific determinants of skeletal muscle mass loss, especially those which are available in everyday medical practice. This study aimed to evaluate the association between appendicular skeletal muscle mass (ASM) and anthropometric, body composition, nutritional, inflammatory, metabolic, and kidney function variables in non-dialysis-dependent CKD men.

**Methods:**

A total of 85 men with CKD and eGFR lower than 60 mL/min/1.73 m^2^ were included in the cross-sectional study: 24 participants with eGFR 59–45 mL/min/1.73 m^2^, 32 individuals with eGFR 44–30 mL/min/1.73 m^2^, and 29 men with eGFR ≤29 mL/min/1.73 m^2^. ASM was estimated by bioimpedance spectroscopy (BIS) with the use of a Body Composition Monitor (BCM). To evaluate ASM from BCM, Lin’s algorithm was used. Among anthropometric parameters, height, weight, and body mass index (BMI) were measured. Serum laboratory measurements were grouped into kidney function, nutritional, inflammatory, and metabolic parameters.

**Results:**

ASM was significantly associated with anthropometric and body composition variables. According to the anthropometric parameters, ASM correlated positively with weight, height, and BMI (*p* < 0.001 and *r* = 0.913, *p* < 0.001 and *r* = 0.560, and *p* < 0.001 and *r* = 0.737, respectively). Among body composition variables, ASM correlated significantly and positively with lean tissue mass (LTM) (*p* < 0.001, *r* = 0.746), lean tissue index (LTI) (*p* < 0.001, *r* = 0.609), fat mass (*p* < 0.001, *r* = 0.489), and fat tissue index (FTI) (*p* < 0.001, *r* = 0.358). No other statistically significant correlation was found between ASM and kidney, nutritional, metabolic, and inflammatory variables.

**Conclusion:**

In male patients with CKD stages G3–G5 not treated with dialysis, ASM correlates significantly and positively with anthropometric and body composition parameters such as weight, height, BMI, LTM, LTI, fat mass, and FTI. We did not observe such relationship between ASM and kidney function, nutritional, metabolic, and inflammatory variables.

## Introduction

Muscle atrophy is one of the complications of chronic kidney disease (CKD) and is highly prevalent in this group of patients. Decreased skeletal muscle mass in CKD leads to muscle strength reduction, the loss of patients’ functional independence, and low physical activity. On the other hand, sedentary lifestyle enhances muscle wasting and muscle atrophy. Reduced skeletal muscle mass is associated with increased morbidity, higher number of hospitalizations, and elevated mortality rate (1). Muscle atrophy in CKD is caused by an imbalance between anabolic and catabolic processes in muscle tissue which is in turn the result of numerous derangements such as metabolic acidosis, increased low-grade inflammatory state, insulin resistance, hormonal alterations, changes in adipocytokine profile, and the impaired mechanisms of hypothalamic appetite regulation (2). Decreased muscle mass deepens with the progression of kidney function impairment and may be diagnosed in 9.5% to even up to 73.4% of patients requiring hemodialysis treatment (3, 4).

Muscle mass-related measurements such as mid-arm muscle circumference (MAMC) or numerous tools such as malnutrition-inflammation score (MIS) have been used in the evaluation of nutritional status in CKD for many years (5, 6). Although accurate assessment of skeletal muscle mass is not included in the diagnostic process of protein-energy wasting (PEW), cachexia and frailty which reflect malnutrition, it is one of the components of sarcopenia (7, 8).

The presence of reduced skeletal muscle mass and/or impaired muscle quantity, along with low muscle strength, enables to diagnose sarcopenia according to the updated guidelines of the European Working Group on Sarcopenia in Older People (EWGSOP2) 2018 (9). Due to the lack of specific assessment criteria for patients with a kidney function decrease, the EWGSOP2 criteria are also used in research studies in the CKD population. Sarcopenia is nowadays thought to be associated with increased morbidity and mortality (10). The prevalence of sarcopenia in CKD increases with the progression of kidney function decrease and ranges from 5.9 to 55% in non-dialysis-dependent CKD patients to up to 63.3% in those undergoing hemodialysis treatments (3, 11). However, there still exist numerous patients with CKD who have normal muscle quality or quantity but low muscle strength, and conversely, those with proper muscle strength but decreased muscle mass. These patients should not be classified as sarcopenic, but rather as pre-sarcopenic individuals (12, 13). It is advisable to overtake the sarcopenia development in CKD population by diagnosing those two compartments separately which could enable to implement proper therapeutic procedures as well as lifestyle and nutritional modification.

There are several techniques which evaluate muscle mass including magnetic resonance imaging (MRI), computed tomography (CT), and dual-energy X-ray absorptiometry (DXA) which is also used to measure appendicular skeletal muscle mass (ASM) (9). A simple, easy to use and not expensive method that also allows to evaluate muscle mass is bioelectrical impedance analysis (BIA). BIA uses a current with a single frequency of 50 kHz, evaluates the overall electrical conductivity of the body, and provides estimation for total body water, fat-free mass (FFM), and fat mass (FM). The main disadvantage of this method is the possibility of measurement imprecision in case of hydration disturbances, which are frequent in the state of kidney failure. A more accurate alternative to BIA is multifrequency bioimpedance spectroscopy (BIS). This technique uses various current frequencies (ranging from 1 to 1,000 kHz) to determine total body water (TBW), as well as extracellular water (ECW) and intracellular water (ICW), lean tissue mass (LTM), and adipose tissue mass (ATM) (14). BIS has greater accuracy than BIA in evaluating hydration status, and thus, it is preferably used in CKD patients (15). By employing various different equations, bioimpedance analysis assesses ASM (16–18).

The concentration changes of serum parameters which may be simply evaluated in medical everyday practice were observed to be associated with the loss of muscle mass, for example, plasma albumin was found to be related positively to skeletal muscle mass in the general population of elderly (19). Increased subclinical inflammatory status which is observed in CKD patients also contributes to skeletal muscle atrophy (20). In addition, hormonal alterations including the derangement of testosterone, growth hormone (GH), and insulin in patients with a kidney function decrease take a significant part in lowering muscle mass (21). Moreover, the deficiency of vitamin D in CKD also contributes to muscle atrophy in this group (22).

Because decreased skeletal muscle mass in CKD is associated with increased morbidity and mortality, it is desirable to understand the mechanisms involved in the loss of ASM and to search for relationships between ASM and multivarious parameters to find potential determinants of skeletal muscle mass loss, especially those which are available in everyday medical practice. Only few studies evaluated the relationships between ASM and different parameters in CKD patients, indicating the need for such analyses (23, 24).

This study aimed to evaluate the association between ASM and anthropometric, body composition, nutritional, inflammatory, metabolic, and kidney function variables in non-dialysis-dependent CKD men.

## Materials and methods

### Study design

We performed a cross-sectional study which included male patients with CKD not treated with dialysis.

### Patients

Participants who were recruited for the study remained under care and visited Nephrological Outpatient Clinic of the Military Institute of Medicine-National Research Institute in Warsaw, Poland, for a routine check-up. All patients were enrolled to the study between November 2018 and February 2020. A total of 85 men with CKD and eGFR lower than 60 mL/min/1.73 m^2^ were included. Each participant signed an informed consent. Participants were classified into three groups based on the value of eGFR according to KDIGO 2012 Clinical Practice Guideline for the Evaluation and Management of Chronic Kidney Disease: patients with eGFR 59–45 mL/min/1.73 m^2^—stage G3a, participants with eGFR 30–44 mL/min/1.73 m^2^—stage G3b, and individuals with eGFR ≤29 mL/min/1.73 m^2^—stage G4–G5 (25). The inclusion criteria were age between 18 and 80 years and eGFR <60 mL/min/1.73 m^2^. The exclusion criteria were as follows: the lack of agreement to take part in the study, clinical signs of infection, the presence of metal parts in the body, and renal replacement therapy or its requirement within the following 3 months.

### Studied variables

#### Muscle mass

Muscle quantity was measured by BIS with the use of a Body Composition Monitor (BCM, Fresenius Medical Care) after 12 h of fasting. Patients were asked to avoid physical exertion and alcohol consumption the day before the examination. Patients remained in a supine position after a 5 min rest with electrodes placed on the one hand and one foot in a tetrapolar configuration. To evaluate muscle mass from BCM, we used the Lin’s algorithm, which derived a formula for ASM estimation based on parameters obtained from bioimpedance spectroscopy and the sum of fat-free soft tissues in the arms and legs assessed from DXA (16).


$$\begin{array}{l} {ASM}_{BCM}=-1.838+0.395\times \mathrm{total}\;\mathrm{body}\;\mathrm{water}+\\ \;0.105\times \mathrm{body}\;\mathrm{weight}-0.026\times \mathrm{age}+\\ \;1.231\left(\mathrm{if}\;\mathrm{male}\right) \end{array}$$

Lin’s prediction model was originally based on the observations of the group of Taiwanese patients with CKD in which Pearson’s correlation coefficient between estimated ASM_BCM_ and ASM_DXA_ was *r* = 0.953 (*p* < 0.001). The limit of agreement of model-derived ASM compared to DXA-derived ASM was 0.098 ± 2.440 kg in Bland–Altman analysis.

We applied Lin’s formula to the independent sample of 109 Polish CKD patients (eGFR <30 mL/min/1.73 m^2^) in which both DXA and BCM measurements were collected to validate Lin’s prediction model. The study design has been previously described (26). The Pearson correlation coefficient between estimated ASM_BCM_ and ASM_DXA_ was 0.954 (*p* < 0.001), and the limit of agreement in Bland–Altman analysis of model-derived ASM compared to DXA-derived ASM was 0.949 ± 2.698 kg.

#### Anthropometric parameters

Among anthropometric parameters, we measured height, weight, and body mass index (BMI).

Height was measured with the use of calibrated stadiometer. The patient was standing straight and barefoot with heels joined on a stadiometer, lightly touched the chin to the neck, joined the heels, and pressed the entire body against the stadiometer. The measurement device of the stadiometer lightly touched the top of the head called the vertex.

The weight of patients was measured in a standing position with the use of calibrated weight scale.

BMI was calculated by dividing patient weight in kilograms by the square of height in meters.

#### Body composition measurements

Body compositions including lean tissue mass (LTM), lean tissue index (LTI), fat mass (Fat), relative fat (Rel Fat), fat tissue index (FTI), overhydration (OH), and relative overhydration (Rel OH) were measured by bioimpedance spectroscopy with the use of a Body Composition Monitor (Fresenius Medical Care, Bad Homburg, Germany). LTI was calculated as lean tissue mass in kilograms divided by height in squared meters, and FTI was calculated as fat mass in kilograms divided by height in squared meters.

#### Blood sample measurements

Blood samples for laboratory measurements were taken after an overnight fast and were analyzed in the local Department of Laboratory Diagnostics. Laboratory measurements were grouped into kidney function parameters: serum creatinine, estimated glomerular filtration rate (eGFR), and urea; nutritional parameters: serum albumin, total cholesterol, hemoglobin, and vitamin D; inflammatory parameters: C-reactive protein (CRP) and tumor necrosis factor-alpha (TNF-alpha); metabolic parameters: low-density lipoprotein cholesterol (LDL), high-density lipoprotein cholesterol (HDL), triglycerides (TG), serum glucose, hemoglobin A1c (HgbA1c), insulin, and homeostatic model assessment of insulin resistance (HOMA-IR). Serum creatinine concentrations were analyzed using Jaffe method (Gen.2, Roche Diagnostics GmbH, Rotkreuz, Switzerland) and serum urea levels using urease kinetic test (Cobas c501, Roche Diagnostics, GmbH, Rotkreuz, Switzerland). Plasma albumin levels were measured with the use of the BCP Albumin Assay Kit (Roche Diagnostics GmbH, Rotkreuz, Switzerland), and serum concentration of CRP was determined by a nephelometry assay (BN^™^ II System Siemens). Samples for measuring TNF-alpha levels were assessed using the Bio-Plex MAGPIX (Luminex Corporation, Austin, TX, United States).

#### The assessment of eGFR

eGFR (mL/min per 1.73 m^2^) was calculated according to the short Modification of Diet in Renal Disease (MDRD) formula: GFR in mL/min per 1.73 m^2^ = 175 × SerumCr ^(−1.154)^ × age ^(−0.203)^ × 1.212 (if patient is black).

### Ethics consideration

The study was conducted in accordance with the Declaration of Helsinki. The study protocol was accepted by the local ethics committee—Bioethics Committee in Military Institute of Medicine-National Research Institute in Warsaw, Poland, IRB acceptance number 120/WIM/2018, obtained 22 August 2018.

### Statistical analysis

The results are presented as means ± standard deviations (SD) for normally distributed data or medians and interquartile ranges (25th–75th percentiles) for skewed distributions. The Kolmogorov–Smirnov test was used to evaluate distributions for normality. One-way ANOVA with trend analysis or Jonckheere–Terpstra test was applied for trend evaluations across CKD stages, depending on assumption regarding distributions. For correlation analysis, Pearson’s correlation coefficients and partial correlation coefficients were calculated. A *p*-value of <0.05 was considered to be statistically significant. Statistical analysis was performed using IBM SPSS v.25.0 software (SPSS Inc., Chicago, IL, United States).

## Results

The study sample consisted of 85 male patients: 24 participants with eGFR 59–45 mL/min/1.73 m^2^ (G3a, 28.2%), 32 individuals with eGFR 44–30 mL/min/1.73 m^2^ (G3b, 37.6%), and 29 men with eGFR ≤29 mL/min/1.73 m^2^ (G4–5, 34.1%). The mean age of all participants was 64 years. Patients with eGFR <29 mL/min/1.73 m^2^ were younger than individuals in stages G3b and G3a of CKD. We did not observe significant differences between the three groups of patients according to ASM and anthropometric variables. Men with stage G4-5 of CKD presented significantly higher fluid overload (*p* = 0.006, *p* = 0.008) (Table 1).

**Table 1:** Anthropometric and body composition characteristics of the studied population.

	Total (*n* = 85)	CKD stage			*p*_trend_
		G3a (*n* = 24)	G3b (*n* = 32)	G4–5 (*n* = 29)	
ASM (BIS) [kg]	24.88 ± 4.50	25.18 ± 3.61	24.31 ± 4.69	25.27 ± 5.02	0.897
Age [years]	63.93 ± 10.56	65.79 ± 8.56	66.56 ± 11.50	59.48 ± 9.83	**0.021**
Anthropometric parameters					
Weight [kg]	89.41 ± 16.64	90.43 ± 14.25	89.85 ± 16.87	88.08 ± 18.61	0.606
Height [m]	1.75 ± 0.07	1.75 ± 0.06	1.74 ± 0.08	1.75 ± 0.07	0.729
BMI [kg/m^2^]	29.27 ± 4.82	29.54 ± 4.12	29.77 ± 5.27	28.50 ± 4.91	0.414
Body composition parameters					
LTM [kg]	51.00 ± 10.33	52.60 ± 7.94	48.14 ± 10.13	52.83 ± 11.82	0.843
LTI [kg/m^2^]	16.67 ± 2.94	17.16 ± 2.06	15.90 ± 3.08	17.11 ± 3.31	0.961
Fat [kg]	27.52 ± 10.99	27.49 ± 9.57	30.05 ± 10.27	24.76 ± 12.47	0.319
Rel Fat [%]	30.05 ± 8.40	29.80 ± 6.63	32.83 ± 8.00	27.19 ± 9.34	0.197
FTI [kg/m^2^]	12.29 ± 4.84	12.24 ± 4.22	13.60 ± 4.78	10.89 ± 5.14	0.258
OH [L]	0.20 ± 1.96	−0.42 ± 1.27	−0.07 ± 1.63	1.02 ± 2.47	**0.006**
Rel OH [%]	0.46 ± 9.14	−2.34 ± 6.63	−0.78 ± 8.00	4.14 ± 11.01	**0.008**

ASM (BIS), appendicular skeletal muscle mass measured by bioimpedance spectroscopy; BMI, body mass index; LTM, lean tissue mass; LTI, lean tissue index; Rel Fat, relative fat; FTI, fat tissue index; OH, overhydration; Rel OH, relative overhydration; eGFR, estimated glomerular filtration rate; *p*_trend_-values < 0.05 are marked in bold.

All patients had serum creatinine concentrations above 1.2 mg/dL, and 70.2% of participants had plasma urea levels above 55 mg/dL. Serum albumin concentrations lower than 3.9 g/dL were observed in 6.0% of all participants. Plasma hemoglobin was lower than 11 g/dL in 14.1% of patients, and total cholesterol was lower than 200 mg/dL in 30.6% of participants. Serum vitamin D below 30 ng/mL was observed in 89.1% patients. All participants had plasma CRP more than or equal to 0.1 mg/dL. Serum LDL concentrations were above 130 mg/dL in 29.4% of patients, 21.1% of participants had plasma HDL lower than 35 mg/dL, and serum TG levels above 165 mg/dL were found in 43.5% of individuals. Plasma glucose concentrations were above 106 mg/dL in 35.7% of patients. Insulin levels were above 24.9 μIU/mL in 29.4% of individuals, and 72.6% of patients had HOMA-IR more than or equal to 2.0. HgbA1c was observed to be above 6.5% in 23.5% of participants.

Participants with lower eGFR had significantly lower serum albumin, plasma hemoglobin, and serum vitamin D concentrations (*p*_trend_ = 0.019, *p*_trend_ < 0.001, and *p*_trend_ = 0.038, respectively) and higher TNF-alpha plasma levels (*p*_trend_ = 0.001) (Table 2).

**Table 2:** Characteristics of the studied population according to kidney function, nutritional, inflammatory, and clinical variables.

	Total (*n* = 85)	CKD stage			*p*_trend_
		G3a (*n* = 24)	G3b (*n* = 32)	G4–5 (*n* = 29)	
Kidney function parameters					
Serum creatinine [mg/dL]	1.90 (1.50–2.65)	1.40 (1.33–1.50)	1.90 (1.70–2.00)	3.20 (2.60–4.00)	**<0.001**
eGFR [mL/min/1.73 m^2^]	37.00 ± 14.28	53.75 ± 3.73	39.34 ± 4.94	20.55 ± 6.79	**<0.001**
Urea [mg/dL]	65.50 (52.00–96.75)	47.00 (43.00–55.75)	63.00 (56.00–80.00)	116.00 (82.50–135.50)	**<0.001**
Nutritional parameters					
Serum albumin [g/dL]	4.40 (4.10–4.60)	4.50 (4.40–4.80)	4.45 (4.13–4.60)	4.30 (4.10–4.50)	**0.019**
Serum hemoglobin [g/dL]	13.25 ± 1.94	14.51 ± 1.60	13.39 ± 1.70	12.05 ± 1.76	**<0.001**
Total cholesterol [mg/dL]	166.00 (142.50–208.00)	167.50 (147.75–233.00)	169.50 (142.00–206.75)	160.00 (135.00–196.00)	0.297
Vitamin D [ng/mL]	22.59 ± 10.38	26.93 ± 13.03	21.11 ± 7.92	20.72 ± 9.66	**0.038**
Inflammatory parameters					
CRP [mg/dL]	0.20 (0.10–0.50)	0.20 (0.10–0.30)	0.30 (0.10–0.60)	0.30 (0.10–0.45)	0.277
TNF-alpha [pg/ml]	4.36 (3.47–5.70)	4.05 (3.00–5.07)	4.24 (3.33–4.81)	5.38 (4.34–6.84)	**0.001**
Metabolic parameters					
LDL [mg/dL]	107. 00 (79.50–142.00)	109.50 (85.50–158.25)	110.00 (80.25–142.25)	104.00 (68.50–128.50)	0.201
HDL [mg/dL]	42. 00 (35.00–53.00)	44.50 (38.25–55.50)	39.50 (32.75–50.75)	41.00 (33.50–53.50)	0.296
TG [mg/dL]	145.00 (110.00–220.00)	128.50 (95.00–288.50)	144.50 (119.50–198.00)	163.00 (117.00–232.00)	0.369
Serum glucose [mg/dL]	95.00 (82.00–129.50)	90.00 (79.00–99.00)	105.50 (85.25–146.50)	98.00 (84.50–143.50)	0.070
Insulin [uIU/mL]	14.10 (7.75–27.90)	14.55 (8.73–28.58)	17.20 (6.85–32.18)	12.80 (8.30–20.20)	0.249
HOMA-IR	3.79 (1.92–7.43)	3.70 (1.65–7.04)	4.61 (1.96–8.54)	2.72 (2.04–7.32)	0.708
HgbA1c [%]	5.70 (5.30–6.40)	5.55 (5.20–6.00)	5.85 (5.33–6.98)	5.80 (5.25–7.05)	0.300

CRP, C-reactive protein; TNF-alpha, tumor necrosis factor-alpha; LDL, low-density lipoprotein cholesterol; HDL, high-density lipoprotein cholesterol; TG, triglyceride; HOMA-IR, homeostatic model assessment of insulin resistance; HgbA1c, hemoglobin A1c; *p*_trend_-values < 0.05 are marked in bold.

**Figure 1:**
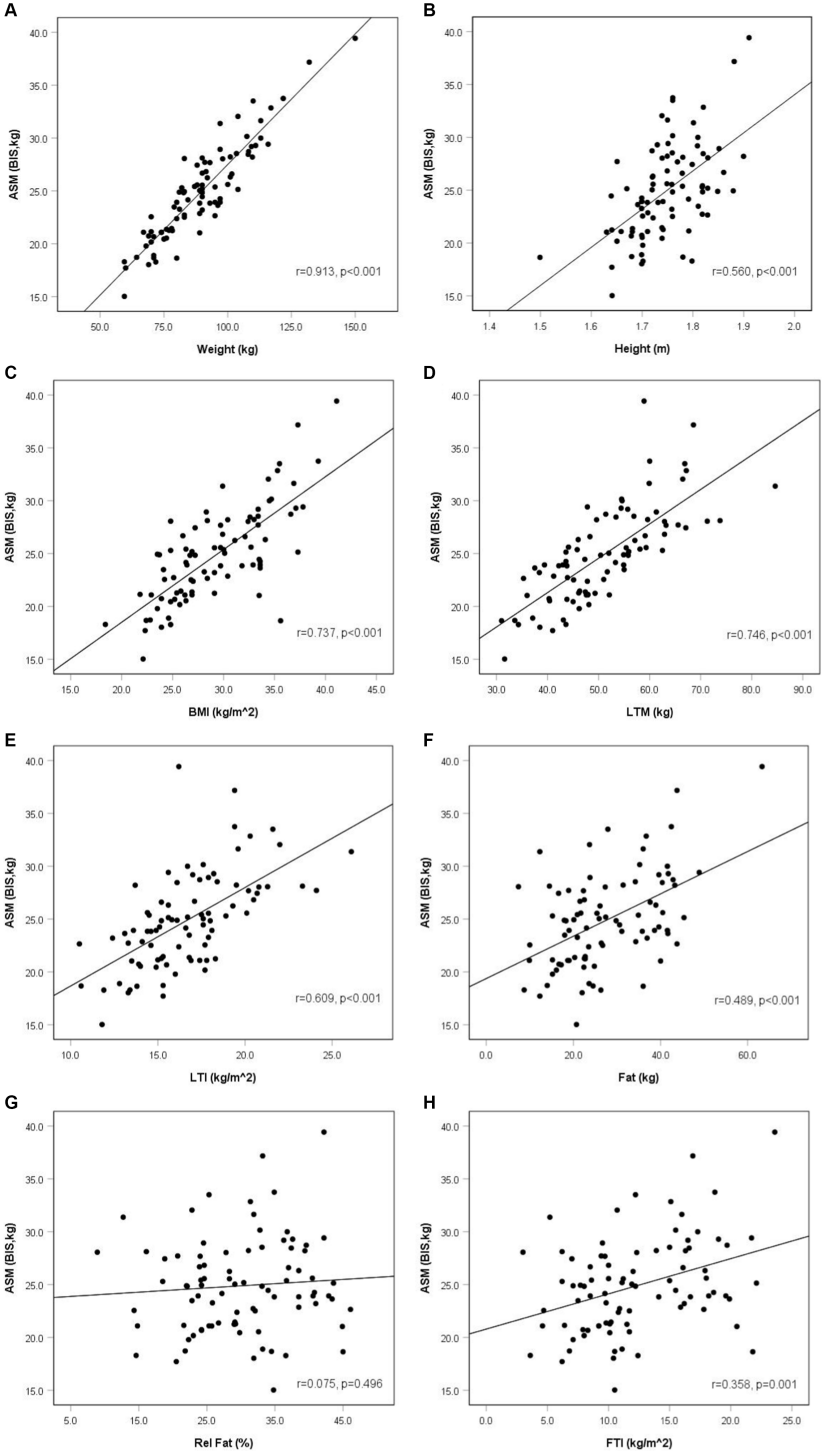
Appendicular skeletal muscle mass and anthropometric and body composition parameters in non-dialysis dependent chronic kidney disease men (*r*—Pearson’s correlation coefficient). Anthropometric and body composition parameters: weight **(A)**, height **(B)**, BMI **(C)**, LTM **(D)**, LTI **(E)**, fat **(F)**, rel fat **(G)**, FTI **(H)**.

We found that older patients had lower ASM (*p* = 0.013). The analysis of correlation between ASM and studied variables revealed that ASM was significantly associated mainly with anthropometric and body composition variables. According to the anthropometric parameters, ASM correlated positively with weight, height, and BMI (*p* < 0.001, *p* < 0.001, and *p* < 0.001, respectively). Among body composition variables, ASM correlated significantly and positively with LTM (*p* < 0.001), LTI (*p* < 0.001), fat mass (*p* < 0.001), and FTI (*p* < 0.001) (Table 3). Among blood measurements, we observed a negative statistically significant relationship between ASM and HDL (*p* < 0.001) and a positive between ASM and TG (*p* = 0.038). However, after adjustment for BMI, using partial correlation analysis, there was neither statistically significant association between HDL and ASM nor between TG and ASM (*r*_partial_ = −0.001, *p* = 0.995 and *r*_partial_ = −0.013, *p* = 0.907, respectively). No other significant correlations were found between ASM and kidney, nutritional, metabolic, and inflammatory variables (Table 3 and Figure 1).

**Table 3:** Pearson’s correlation analysis of ASM with anthropometric, body composition, kidney function, nutritional, metabolic, and inflammatory parameters.

	*r*	*p*
Age [years]	−0.268	**0.013**
**Anthropometric parameters**		
Weight [kg]	0.913	**<0.001**
Height [m]	0.560	**<0.001**
BMI [kg/m^2^]	0.737	**<0.001**
**Body composition parameters**		
LTM [kg]	0.746	**<0.001**
LTI [kg/m^2^]	0.609	**<0.001**
Fat [kg]	0.489	**<0.001**
Rel Fat [%]	0.075	0.496
FTI [kg/m^2^]	0.358	**0.001**
OH [L]	0.095	0.389
Rel OH [%]	0.002	0.982
**Kidney function parameters**		
Serum creatinine [mg/dL]	−0.003	0.977
eGFR [mL/min/1.73 m^2^]	0.066	0.547
Urea [mg/dL]	−0.001	0.991
**Nutritional parameters**		
Serum albumin [g/dL]	−0.070	0.529
Serum hemoglobin [g/dL]	−0.068	0.539
Total cholesterol [mg/dL]	−0.058	0.598
Vitamin D [ng/ml]	−0.087	0.432
**Metabolic parameters**		
LDL [mg/dL]	−0.063	0.568
HDL [mg/dL]	−0.370	**<0.001**
TG [mg/dL]	0.226	**0.038**
Serum glucose [mg/dL]	0.066	0.553
Insulin [uIU/mL]	0.046	0.677
HOMA-IR	0.046	0.979
HgbA1c [%]	0.031	0.779
**Inflammatory parameters**		
CRP [mg/dL]	−0.005	0.962
TNF-alpha [pg/ml]	−0.118	0.284

BMI, body mass index; LTM, lean tissue mass; LTI, lean tissue index; Rel Fat, relative fat; FTI, fat tissue index; OH, overhydration; Rel OH, relative overhydration; eGFR, estimated glomerular filtration rate; LDL, low-density lipoprotein cholesterol; HDL, high-density lipoprotein cholesterol; TG, triglycerides; HOMA-IR, homeostatic model assessment of insulin resistance; HgbA1c, hemoglobin A1c; CRP, C-reactive protein; TNF-alpha, tumor necrosis factor-alpha; *r*-correlation coefficient, *p*-values < 0.05 are marked in bold.

## Discussion

In our study, we found that ASM in CKD men not treated with dialysis with eGFR <60 mL/min/1.73 m^2^ was positively correlated with anthropometric and body composition parameters. ASM was higher in taller, heavier, and obese participants. We did not observe statistically significant correlations between ASM and kidney, nutritional, and inflammatory parameters. Among metabolic parameters after adjustment for BMI, we also did not find a statistically significant association between ASM and TG and between ASM and HDL.

### Anthropometric and body composition parameters

Regarding body composition parameters, ASM was higher not only in participants with higher LTM and LTI but also with increased fat mass and FTI. Such results emphasize the significant role of anthropometric measurements in the group of CKD patients in terms of the necessity to evaluate skeletal muscle mass and the probability of muscle atrophy. Some studies found similar results to ours; however, they analyze muscle mass together with other characteristics. The report of Wang revealed that the MIS in CKD patients stages G3–G5 was negatively correlated with anthropometric parameters such as BMI and MAMC and also with body composition variables such as LTI and FTI (27). The study of Heimbürger, similar to our results, also showed that low-fat mass was an independent factor related to malnutrition in CKD participants (28). These results are similar to ours as we observed that individuals with higher fat mass and FTI had higher ASM. Sánchez-Tocino found the relationship between sarcopenia and nutrition; however, they observed the opposite to our results according to weight—patients with confirmed sarcopenia were heavier and had higher fat mass (29).

As it was noticed previously, muscle atrophy is associated with higher mortality in CKD. The report of Stosovic et al. found that in the group of patients treated with hemodialysis, anthropometric parameters such as the percentage of body fat, mid-arm circumference (MAC), MAMC, and the percentage of body fat calculated from triceps (TSF) were independent predictors of mortality in this group. Increased values of anthropometric parameters were associated with reduced mortality. However, they did not observe the association between BMI and mortality (30). Increased anthropometric and body composition variables along with higher ASM and lower mortality may play a role in the obesity paradox phenomenon observed in CKD. The term “obesity paradox” reflects the reverse epidemiology of obesity which shows that CKD patients with lower BMI or weight loss have higher mortality, and conversely, those with increased BMI or weight gain present better survival. This means that overweight and obesity play a protective role in CKD (31). The report of Lu which analyzed data of over 450 participants with eGFR <60 mL/min/1.73m^2^ observed that BMI <25 kg/m^2^ was associated with worse outcomes, independently of the severity of CKD. In addition, BMI levels below 30 kg/m^2^ were linearly related to higher mortality (32). Ahmandi also presented the results of 10 studies which included nearly 500 participants with CKD stages G3-G5 and revealed that underweight patients had higher mortality rates compared to overweight and obese class I individuals. Moreover, obesity class II and class III were not related to increased mortality (33). Bellafronte assumed that the obesity paradox in CKD may be due to higher lean mass and its protective effect in obese individuals (34). In our report, we found that ASM was significantly and positively associated with BMI, LTM, LTI, as well as fat mass and FTI. Thus, we may assume that simple parameter such as BMI and body composition variables estimated by BIS may be useful to estimate ASM in CKD patients. Moreover, we also observed the positive correlation of height with ASM which is probably associated with higher absolute muscle mass in taller individuals.

### Kidney function parameters

We did not observe a significant correlation of ASM with kidney function parameters such as serum creatinine, eGFR, and urea. Comparable to our results, also in the study of Wang, MIS was not correlated with eGFR (27). The lack of association between ASM and kidney function parameters among patients at more advanced stages of CKD in our study does not indicate that there is no difference in the progress of muscle atrophy between patients at all stages of CKD. On the contrary, the numerous mechanisms that lead to muscle atrophy deepen with the progression of renal failure, being the most severe in end-stage kidney disease patients treated with dialysis (35). However, the rate of muscle mass loss varies among patients at different stages of kidney function decrease and that is probably why the simple correlations between ASM in our study or MIS in the report of Wang and kidney function parameters were not observed (27). In addition, the meta-analysis of Duarte revealed that the prevalence of sarcopenia in CKD patients did not differ among stages of kidney function decrease which also indicate that the severity of kidney failure is not the significant determinant of muscle mass loss (36).

### Nutritional and metabolic parameters

We did not find a statistically significant relationship between ASM and serum albumin, nor between ASM and hemoglobin. The study by Wang found opposite to ours results as they observed that MIS was strongly and negatively associated with serum nutritional parameters such as plasma albumin and hemoglobin (27, 37, 38). The evaluation of nutritional status in CKD includes the measurement of serum albumin levels. Decreased plasma albumin is one of the criteria of protein-energy wasting syndrome in CKD (5). However, some studies did not find a relationship between plasma albumin and nutritional status in this population (28, 29, 39). The study of Evans confirmed that albumin is a poor nutritional marker (40). We also did not observe a significant correlation between ASM and serum albumin. The probable reason of it is that albumin plasma levels depend on hepatic function and play a role as an acute-phase protein. Thus, serum albumin is rather a poor nutritional marker in CKD. Sánchez-Tocino did not find the relationship between sarcopenia and serum albumin, but the association between sarcopenia and serum total cholesterol was positive (29). Some studies in the general population observed the positive association between the advancement of dyslipidemia and decreased skeletal muscle mass (41, 42). In our study, we did not report the relationship between ASM and total cholesterol and between ASM and LDL, but we found a significant negative zero-order correlation between ASM and HDL and a positive between ASM and TG. After adjustment for BMI, these last two associations disappeared since BMI was a confounding variable in the relationships between ASM and HDL and between ASM and TG.

### Inflammatory parameters

We did not find the association between ASM and CRP and between ASM and TNF-alpha. Wang also did not observe statistically significant correlations between MIS and inflammatory parameters, similar to our results (27). However, the report of Heimbürger showed that high serum CRP is an independent determinant of malnutrition in CKD, contrary to our study (28). Different results concerning the impact of inflammatory cytokines on muscle mass may be related to differences between the studied populations, the amount of skeletal muscle mass, and inflammatory cytokines’ synthesis; despite the fact that inflammatory cytokines act through numerous mechanisms, they activate the nuclear transcription factor-kappa B (NFκB) pathway, raise myostatin expression, and impair the hypothalamic response to appetite-regulating hormones (43, 44).

The limitation of our study is its relatively small sample size and cross-sectional design, not allowing for inferencing about causal relationships. Future prospective cohort studies would allow to observe the dynamics of ASM changes against other parameter fluctuations in CKD patients. Moreover, a larger number of participants would enable a more detailed and more comprehensive analysis. In addition, our study is restricted to one gender—male. The study with female participants would also let compare the results between men and women.

We may conclude that in male patients with CKD stages G3–G5 not treated with dialysis, ASM correlates significantly and positively with anthropometric and body composition parameters such as weight, height, BMI, LTM, LTI, fat mass, and FTI. We did not observe such relationship between ASM and kidney function, nutritional, metabolic, and inflammatory variables.

## Data availability statement

The raw data supporting the conclusions of this article will be made available by the authors, without undue reservation.

## Ethics statement

The studies involving humans were approved by Bioethics Committee in Military Institute of Medicine—National Research Institute in Warsaw. The studies were conducted in accordance with the local legislation and institutional requirements. The participants provided their written informed consent to participate in this study.

## Author contributions

KR: Conceptualization, Data curation, Investigation, Methodology, Writing – original draft. KS: Conceptualization, Data curation, Formal analysis, Methodology, Writing – review & editing. AR: Investigation, Methodology, Project administration, Writing – review & editing. RT: Writing – review & editing. SN: Funding acquisition, Supervision, Writing – review & editing.
